# Prediction of protein structures, functions and interactions using the IntFOLD7, MultiFOLD and ModFOLDdock servers

**DOI:** 10.1093/nar/gkad297

**Published:** 2023-04-27

**Authors:** Liam J McGuffin, Nicholas S Edmunds, Ahmet G Genc, Shuaa M A Alharbi, Bajuna R Salehe, Recep Adiyaman

**Affiliations:** School of Biological Sciences, University of Reading, Whiteknights, ReadingRG6 6EX, UK; School of Biological Sciences, University of Reading, Whiteknights, ReadingRG6 6EX, UK; School of Biological Sciences, University of Reading, Whiteknights, ReadingRG6 6EX, UK; School of Biological Sciences, University of Reading, Whiteknights, ReadingRG6 6EX, UK; School of Biological Sciences, University of Reading, Whiteknights, ReadingRG6 6EX, UK; School of Biological Sciences, University of Reading, Whiteknights, ReadingRG6 6EX, UK

## Abstract

The IntFOLD server based at the University of Reading has been a leading method over the past decade in providing free access to accurate prediction of protein structures and functions. In a post-AlphaFold2 world, accurate models of tertiary structures are widely available for even more protein targets, so there has been a refocus in the prediction community towards the accurate modelling of protein-ligand interactions as well as modelling quaternary structure assemblies. In this paper, we describe the latest improvements to IntFOLD, which maintains its competitive structure prediction performance by including the latest deep learning methods while also integrating accurate model quality estimates and 3D models of protein-ligand interactions. Furthermore, we also introduce our two new server methods: MultiFOLD for accurately modelling both tertiary and quaternary structures, with performance which has been independently verified to outperform the standard AlphaFold2 methods, and ModFOLDdock, which provides world-leading quality estimates for quaternary structure models. The IntFOLD7, MultiFOLD and ModFOLDdock servers are available at: https://www.reading.ac.uk/bioinf/.

## Graphical Abstract

**Graphical Abstract:**
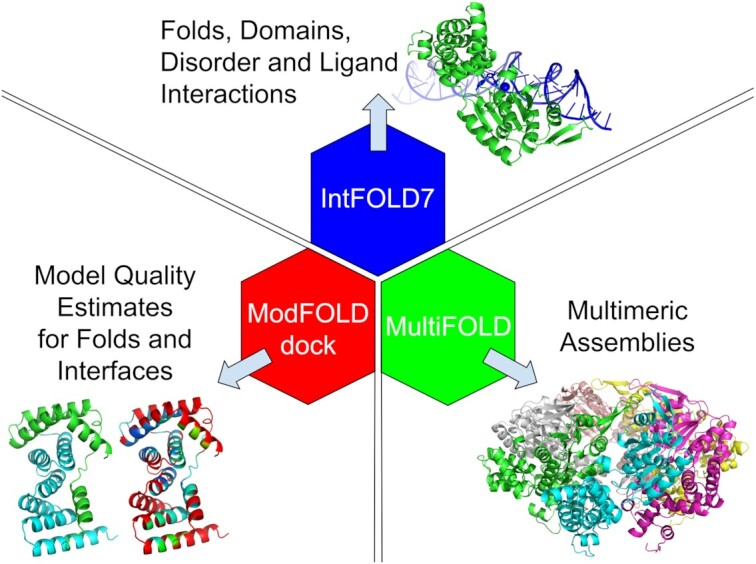
Prediction of protein, structures, functions and interactions at the University of Reading using the IntFOLD7, MultFOLD and ModFOLDdock servers.

## INTRODUCTION

The routine use of highly accurate predicted protein tertiary structure models by life scientists continues to grow, particularly now that we are in a post-AlphaFold2 (1) world. However, the protein structure prediction community continues to face several challenges, including the accurate 3D modelling of protein-ligand and protein-protein interactions. Over the past decade, the IntFOLD server (2–6), based at the University of Reading has been a leading resource providing freely accessible high-quality 3D models with integrated quality estimates, domain and disorder predictions, and models of protein-ligand interactions. The performance of every version of IntFOLD has been independently benchmarked over the successive Critical Assessment of Techniques for Protein Structure Prediction (7) (CASP) experiments and by the continuous CAMEO (8) project. IntFOLD performance has been continually improved and, as a result, server usage has continued to grow year-on-year (Supplementary Figure S1). By the end of 2022, IntFOLD had served ∼25 000 unique users and completed ∼315 000 jobs.

IntFOLD has undergone major modifications to the underlying component methods, which have led to significant performance gains since our last paper describing the server, in 2019 (6). IntFOLD7 integrates improved independent model quality estimates from our latest version of ModFOLD (9–12), which has been retrained using the latest model datasets with recalibrated p-values. Additionally, IntFOLD7 integrates 3D modelling using both trRosetta2 (13) and LocalColabFold (14), allowing it to maintain its competitiveness with the latest deep learning methods.

In the CASP15 experiment, there was an additional focus not only on modelling protein-ligand interactions, such as those provided by the IntFOLD7 server but also on modelling protein-protein interactions. In light of this, we have also developed our two new complimentary servers to specifically address the problems of both modelling quaternary structures (MultiFOLD) and scoring the resulting multimeric models (ModFOLDdock).

MultiFOLD was found to be highly competitive in the CASP15 experiment, ranking among the top ten servers on both the regular monomeric and the multimeric targets. Furthermore, ModFOLDdock was found to be a leading server for estimating the quality of the quaternary structure models. Scoring the quality of 3D models has always been a critical aspect of protein structure prediction pipelines, however, until now there have been few available methods for reliably and independently scoring modelled protein complexes. ModFOLDdock fulfils this pressing need, which is becoming more pertinent as alternative deep learning multimer modelling methods continue to emerge. Following the rigorous independent evaluation by the CASP15 assessors, we have now made the MultiFOLD and ModFOLDdock servers freely available, providing them with intuitive web interfaces to benefit non-expert predictors in the wider bioscience community.

## MATERIALS AND METHODS

For brevity, below we summarise the core aspects of the methodology behind the IntFOLD7, MultiFOLD and ModFOLDdock servers. For further information and details of their specific parameters, please refer to our previous papers, CASP15 abstracts and presentations (https://predictioncenter.org/casp15/).

### IntFOLD7

The IntFOLD7 server provides a single point of access to an integrated suite of six component methods: IntFOLD-TS, for tertiary structure prediction; ModFOLD (9–12), for model quality estimates; ReFOLD (5,15,16), for refinement; DISOclust (17), for disorder prediction; DomFOLD for domain prediction, and FunFOLD (18,19) for ligand binding site prediction. The focus of our improvements for the IntFOLD7 server built upon our successes from previous versions, which used ModFOLD variants to score and rank models. Thus we integrated scores for global model ranking and local model quality scoring, using our newly improved ModFOLD9 method. The ModFOLD9 protocol builds on that of ModFOLD8 (12) by including 6 new integrated scoring methods (3 new Contact Distance Agreement (CDA) scores (9,12), and the 3 variants of the DeepAccNet (20), which were combined using neural networks to provide quality estimates for each model. In addition, we integrated two state-of-the-art deep-learning methods for tertiary structure modelling: LocalColabFold version 1.0.0 (14) and trRosetta2 (13). The IntFOLD7 server is available at: https://www.reading.ac.uk/bioinf/IntFOLD/.

### MultiFOLD

The MultiFOLD server has three principal stages: 3D structure modelling, scoring, and refinement. The first stage involves the generation of 20 models using two different variants of the LocalColabFold (14) method (1.0.0 and 1.3.0) that use alternative approaches to assembly prediction and different weight sets. In the next stage, these models are ranked using our novel ModFOLDdockR (see below) quality estimation method to identify the top 5. The final stage is to refine the top 5 models, by feeding them back into LocalColabFold as template input files and reprocessing them over several cycles. This iterative assembly, quality scoring and refinement process led to significant improvement of model quality beyond the baseline AlphaFold2-Multimer models. The MultiFOLD server is available at: https://www.reading.ac.uk/bioinf/MultiFOLD/. MultiFOLD is also available as a docker image: https://hub.docker.com/r/mcguffin/multifold.

### ModFOLDdock

The ModFOLDdock server uses a novel hybrid consensus approach for scoring predicted quaternary structures, producing both global and local (interface residue) model quality estimates. There are 3 variants of the ModFOLDdock method which use various combinations of scores, which are calculated using the output from 7 individual scoring methods: DockQJury, QSscoreJury QSscoreOfficialJury, lDDTOfficialJury, voronota-js-voromqa (21), CDA (5,12) and ModFOLDIA. For the DockQJury, QSscoreJury, QSscoreOfficialJury and lDDTOfficialJury scoring methods, pairwise comparisons were made between each quaternary structure model and every other model and then the mean QS (22,23), lDDT (24) or DockQ (25) scores were calculated. The ModFOLDdock and ModFOLDdockR variants required multiple models as inputs. The ModFOLDdock variant was optimised to produce quality estimates which correlate linearly with the observed quality scores, while the ModFOLDdockR variant was designed to produce predicted scores optimised for ranking, i.e. the top-ranked models (top 1) should have higher observed overall accuracy. The ModFOLDdockS variant was designed to be used in single-model mode, whereby sets of reference multimer models were firstly generated from the input sequences using our MultiFOLD method (see above) and then each model was compared individually against the reference set using the seven individual scoring methods described above. The ModFOLDdock server is available at: https://www.reading.ac.uk/bioinf/ModFOLDdock/. ModFOLDdock is also available via the MultiFOLD docker image: https://hub.docker.com/r/mcguffin/multifold.

## RESULTS AND DISCUSSION

### IntFOLD7 server inputs and outputs

The only required input for the IntFOLD7 server is a single amino acid sequence in single letter format. However, optionally users may provide a short name for their prediction job and an email address, which will only be used to provide a notification of the link to their results once their predictions are completed. If users do not wish to provide an email address, then they can simply bookmark the link to the results page for later viewing. The prediction data are presented graphically, and models are viewable interactively in 3D. Machine-readable data are also provided which comply with CASP data standards.

**Figure 1.**
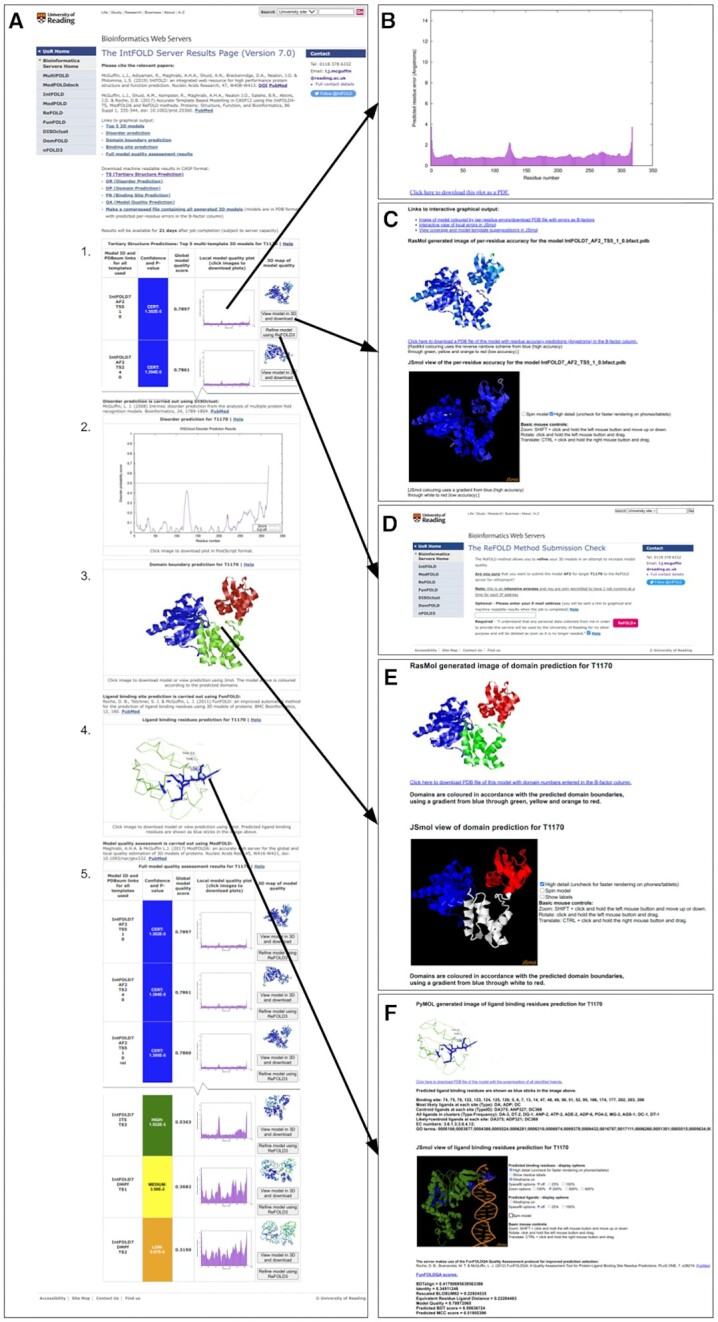
IntFOLD7 server results pages for CASP15 target T1170. (**A**) Graphical output from the main results page showing (from top to bottom): 1. The table with the top 5 selected 3D models and scores (table truncated here to fit); 2. The prediction of natively unstructured/disordered regions; 3. The predicted structural domain boundaries; 4. The ligand binding site prediction; 5. The full model quality rankings for all generated models (table truncated here to fit). The arrows point to the additional pages that are linked when users click on images/buttons on the main page. Users have the option to download coordinates and view the detailed predicted error plots (**B**), they can view model results interactively in 3D coloured by local quality (**C**) and submit individual 3D models for further refinement using ReFOLD (**D**) via simple push buttons. Downloadable coordinates and interactive 3D views of the predicted domain boundaries (**E**) and protein–ligand interactions (**F**) can also be accessed.

Examples of the graphical outputs from the IntFOLD7 server are shown in Figure 1. The output is presented as a single table that graphically summarises all prediction data using thumbnail images of the model accuracy plots and models, links to the template information and colour-coded scoring (Figure 1A). Clicking on the images and buttons on the main page will take the user to further pages with interactive output (Figure 1B–F).

### MultiFOLD server inputs and outputs

The MultiFOLD server requires users to provide their input sequences for the target proteins in FASTA format. Optionally, users may also provide a short name and email address if they wish to receive a notification or they can just bookmark the link to the results pages, which is provided after submission. It is recommended that users also provide stoichiometry information for their complex if this is known. If the stoichiometry is unknown, then MultiFOLD will attempt to predict it from known template structures.

The resulting models of protein complexes can be viewed interactively in 3D directly within a standard desktop or mobile web browser (Supplementary Figure S2). The results table shows the top 5 selected 3D models and is ranked according to decreasing predicted global model quality scores. Each row in the table also provides buttons for colouring models either by chain identifier or predicted local quality, and for downloading models as PDB files. Machine-readable model files are also provided, which comply with the CASP standard TS format.

### ModFOLDdock server inputs and outputs

For the ModFOLDdock server, the user-required inputs for the server are the input sequences for the target proteins provided in FASTA format, the stoichiometry of the target complex, and a single 3D model for evaluation. Optionally, users may upload multiple alternative models, a name for their protein sequence and their email address. Users of the server may also select from three different variants, which use different combinations of component scoring methods optimised for the different facets of the quality estimation problem. Firstly, ModFOLDdock, with predicted global scores that are optimised to provide positive linear correlations with observed scores. Secondly, ModFOLDdockR with global scores optimised for ranking the best models at the top. And finally, ModFOLDdockS, for scoring single models at a time.

The results table ranks models according to the predicted global assembly and interface quality scores (Supplementary Figure S3). As with MultiFOLD, the ranked models of protein complexes can be viewed interactively in 3D directly within a standard desktop or mobile web browser. Each row in the table also provides buttons for colouring models either by chain identifier or, in this case, by the predicted local interface quality. Users may also download models as PDB files with the predicted local interface scores added to the B-factor column. Machine-readable model files are also provided, which comply with the new CASP15 standard QA (QMODE2) format.

### Independent benchmarking

IntFOLD has undergone successive iterative improvements, which have been independently tested in each of the relevant categories of the CASP experiments. IntFOLD7 ranked competitively in the recent CASP15 experiment, particularly in the Interdomain category where it outperformed many leading human predictor groups. In addition, the predictions are also continuously evaluated by the CAMEO project (https://www.cameo3d.org/), where IntFOLD7 ranks as one of the leading freely publicly available methods.

MultiFOLD was highly competitive in CASP15, ranking among the top ten servers on both the regular monomeric and the multimeric targets. Furthermore, it is a pioneering server participating in the new CAMEO category for continuous evaluation of complex modelling.

In CASP15, the ModFOLDdock server was rigorously independently benchmarked and it was found to be a leading approach for the evaluation of quaternary structure models of proteins. ModFOLDdock variants ranked within the top three groups across all assessment metrics in the Estimates of Model Accuracy category and we were invited to speak about the server at the CASP15 conference.

#### CAMEO results summary

The data in Table 1A summarise the results from the CAMEO 3D continuous independent evaluation of IntFOLD7 over 1 year compared with other leading publicly available methods based on common subset analysis. IntFOLD7 performs well compared with other state-of-the-art methods and it is verified to be an improvement over previous versions of the server (IntFOLD3-IntFOLD6, Supplementary Table S1). Table 1B shows that MultiFOLD outperforms the other anonymous participating server (Server76) in the accuracy of modelling complexes according to the latest data.

**Table 1.** Independent benchmarking data from CAMEO. (A) CAMEO 3D data for IntFOLD7 - All Targets, Common Subset Comparison (172 targets) with IntFOLD7 as the reference server - dataset: 1-year [2022–02-04 - 2023–01-28]. Data are from: https://www.cameo3d.org/modeling/. (B) CAMEO ‘Modeling of Structures & Complexes’ Beta common subset comparison for MultiFOLD (Server 1) versus the other reference server (Server76), (118 targets Oligo_lDDT, 28 targets QS-score)—dataset: 11 weeks [2022-10-22 to 2023-01-28] Data are from: https://beta.cameo3d.org/complete-modeling/

A						
	Avg. lDDT		Avg. CAD-score		Avg. lDDT-BS	
Server name	Dif.	Best	Dif.	Best	Dif.	Best
AlphaFoldDB 100	−0.52	82.56	−0.42	78.8	−3.64	82.76
**IntFOLD7**	0	82.04	0	78.39	0	79.11
RoseTTAFold	6.76	75.28	5.45	72.94	8.04	71.07
IntFOLD6-TS	15.31	66.73	12.47	65.92	7.53	71.58
SWISS-MODEL	19.98	62.05	17.19	61.19	10.7	68.41
Phyre2	27.15	54.89	22.59	55.8	15.88	63.23
B						
Server	Sum of Oligo_lDDT			Sum of QS-score		
**MultiFOLD** (Server1)	74.372			15.65		
Server76	69.497			11.636		

#### CASP15 results summary

In the CASP15 experiment, we relied on our IntFOLD7, MultiFOLD and ModFOLDdock servers to help us with our manual predictions. Overall, our McGuffin group ranked sixth in the regular tertiary structure prediction category and 11th in the multimeric target prediction category, according to the default rankings (https://predictioncenter.org/casp15/). Additionally, we made extensive use of the integrated binding site predictions, from the FunFOLD component of IntFOLD7, to submit our manual predictions for the new ligand category.

The IntFOLD7 server itself was one of the top-performing freely accessible servers for tertiary structure predictions, outperforming NBIS-AF2-standard (AlphaFold2) in the interdomain (Supplementary Table S2) and regular categories (Supplementary Tables S3 and S4), along with many renowned servers and human predictor groups.

The MultiFOLD server ranked even higher as the ninth best server group on monomeric targets according to the GDT_TS scores (Supplementary Table S5) and the 8th best server group for multimers by the assessors’ formula (Supplementary Table S6). MultiFOLD outperformed both the baseline NBIS-AF2-standard (AlphaFold2) and NBIS-AF2-multimer (AlphaFold2-Multimer) methods by all measures.

Furthermore, in the CASP15 Estimates of Model Accuracy category, the ModFOLDdock server variants were ranked within the top few groups by all combined measures for the global (fold) scores, the global (interface) scores and the local mode scores (Supplementary Tables S7–S9, respectively).

**Figure 2.**
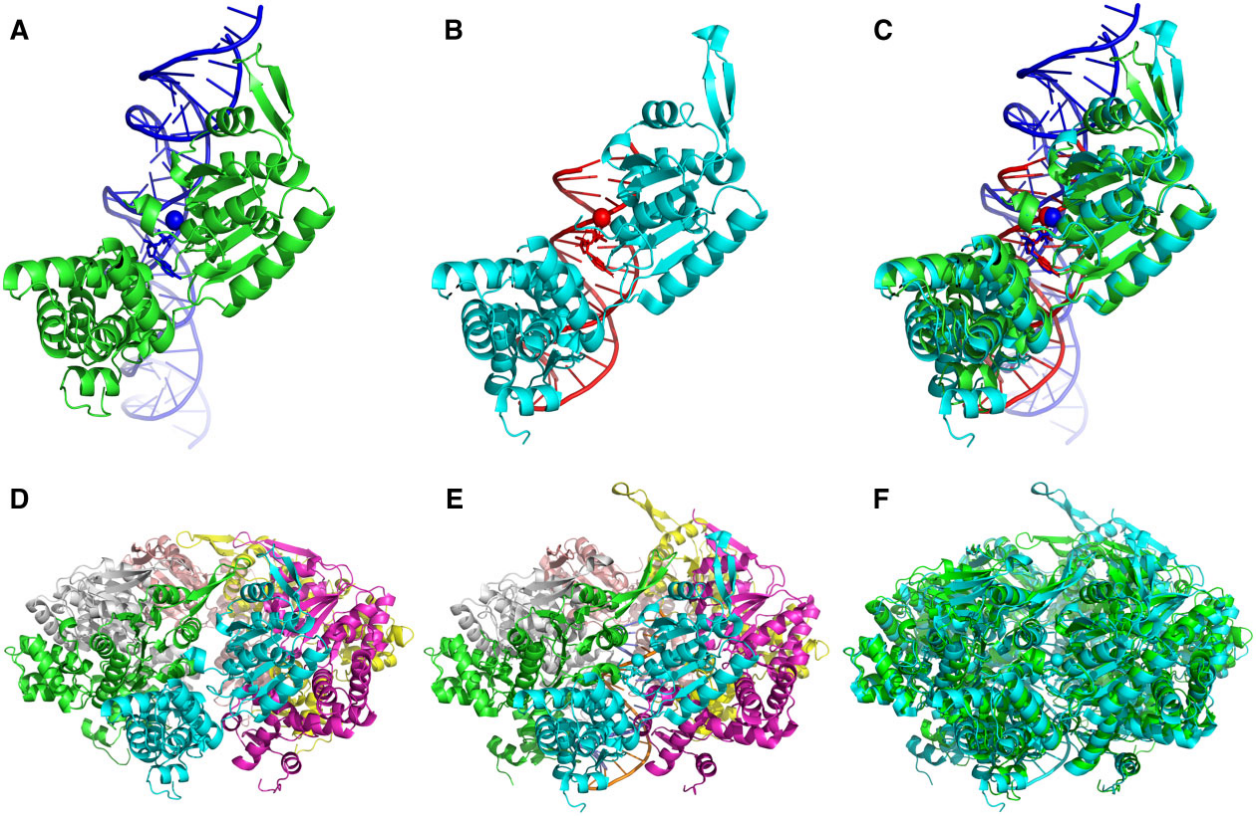
Examples of the IntFOLD7 & MultFOLD server predictions for the hexameric CASP15 target T1170 compared with the native structure (PDB ID 7pbr). All images were rendered using PyMOL (http://www.pymol.org/). (**A**) The top IntFOLD7 3D model of the T1170 tertiary structure (green) with predicted interacting DNA, MG and AGS ligands (blue). (**B**) The native tertiary structure of T1170 (cyan) with interacting DNA, MG and ADP ligands (red). (**C**) Superposition of the predicted and native tertiary structures for T1170 with interacting ligands (domain 1 – GDT_TS = 84.17, lDDT = 0.848; domain 2 – GDT_TS = 100.00, lDDT = 0.885). (**D**) The best MultiFOLD 3D model of the quaternary structure of T1170 coloured by chain ID. (**E**) The native quaternary structure of T1170, coloured by chain ID. (**F**) Superposition of the predicted and native quaternary structures for T1170 (QS = 0.759, Oligo-lDDT = 0.799).

Figure 2 shows an example of IntFOLD7 and MultiFOLD models for CASP15 target T1170 (obtained via the pages shown in Figure 1 and Supplementary Figure S2) compared with the native tertiary and quaternary structures (PDB ID 7pbr). The IntFOLD7 predicted tertiary structure (Figure 2A) is close to the native structure of the subunit shown in Figure 2B. The predicted locations of the DNA and ligand binding sites are also shown to be accurate (Figures 2A & B) and there is a close superposition of the model and native structure (Figure 2C). In addition, the MultiFOLD predicted quaternary structure (Figure 2D) is shown to be accurate compared to the native structure of the hexamer (Figures 2E and F). Further examples of the top MultiFOLD predictions compared with the native structures are shown in (Supplementary Figure S4).

## CONCLUSIONS

The IntFOLD server has maintained its leading-edge performance, providing users with free state-of-the-art 3D modelling with added value from the integrated model quality estimates, protein-ligand interaction predictions, disorder and domain predictions and the option to refine their models further. Furthermore, our new MultiFOLD server is shown to be highly competitive with current quaternary structure modelling methods according to recent blind testing (CASP15) and continuous benchmarks (CAMEO), while our ModFOLDdock server is one of the top few leading methods for estimating the quality of modelled complexes. Following this independent evaluation, we are now also making MultiFOLD and ModFOLDdock freely available, providing intuitive web interfaces to benefit the wider bioscience community.

## DATA AVAILABILITY

The data underlying this article are available in the article and in its online supplementary material.

## Supplementary Material

gkad297_Supplemental_File

## Notes

Present address: Bajuna R. Salehe, Department of Genetics, University of Cambridge, Cambridge CB2 3EH, UK.
