# Supplementary material for: Prediction of protein structures, functions and interactions using the IntFOLD7, MultiFOLD and ModFOLDdock servers
Source: Nucleic Acids Res. 2023 Apr 27;51(W1):W274–80. doi: 10.1093/nar/gkad297 (PMC10320135; doi:10.1093/nar/gkad297)
Supplement: gkad297_Supplemental_File [file gkad297_supplemental_file.pdf]

Supplementary Figure 1. Growth in usage of the IntFOLD server. Extrapolated growth in (red curves) based on historical IntFOLD server data (blue points).

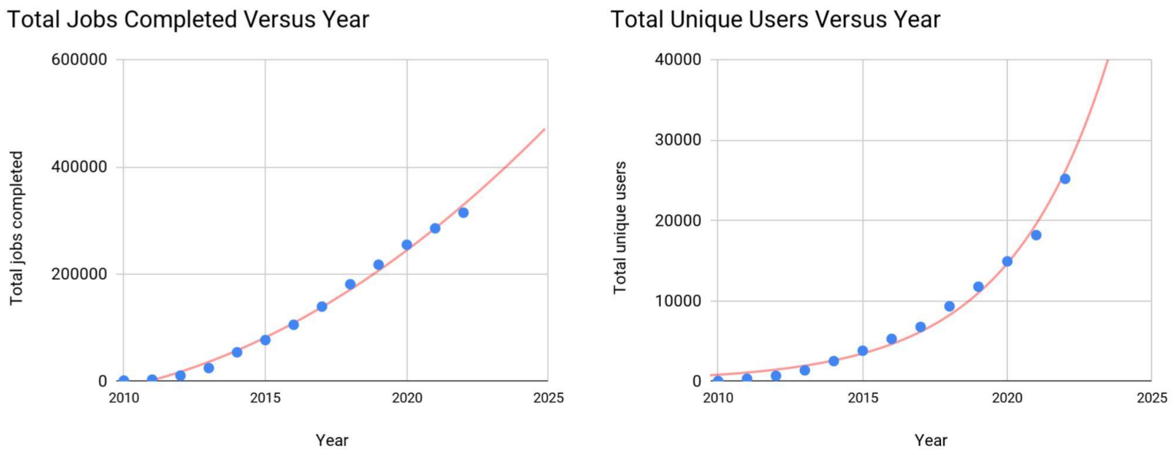

Supplementary Figure 2. MultiFOLD server results pages for CASP15 target T1170 with stoichiometry A6. (A) The top model is coloured by chain identifier which can be selected using the “Show Chains” button. (B) The top model is coloured by predicted local model quality (the per-residue plDDT scores).

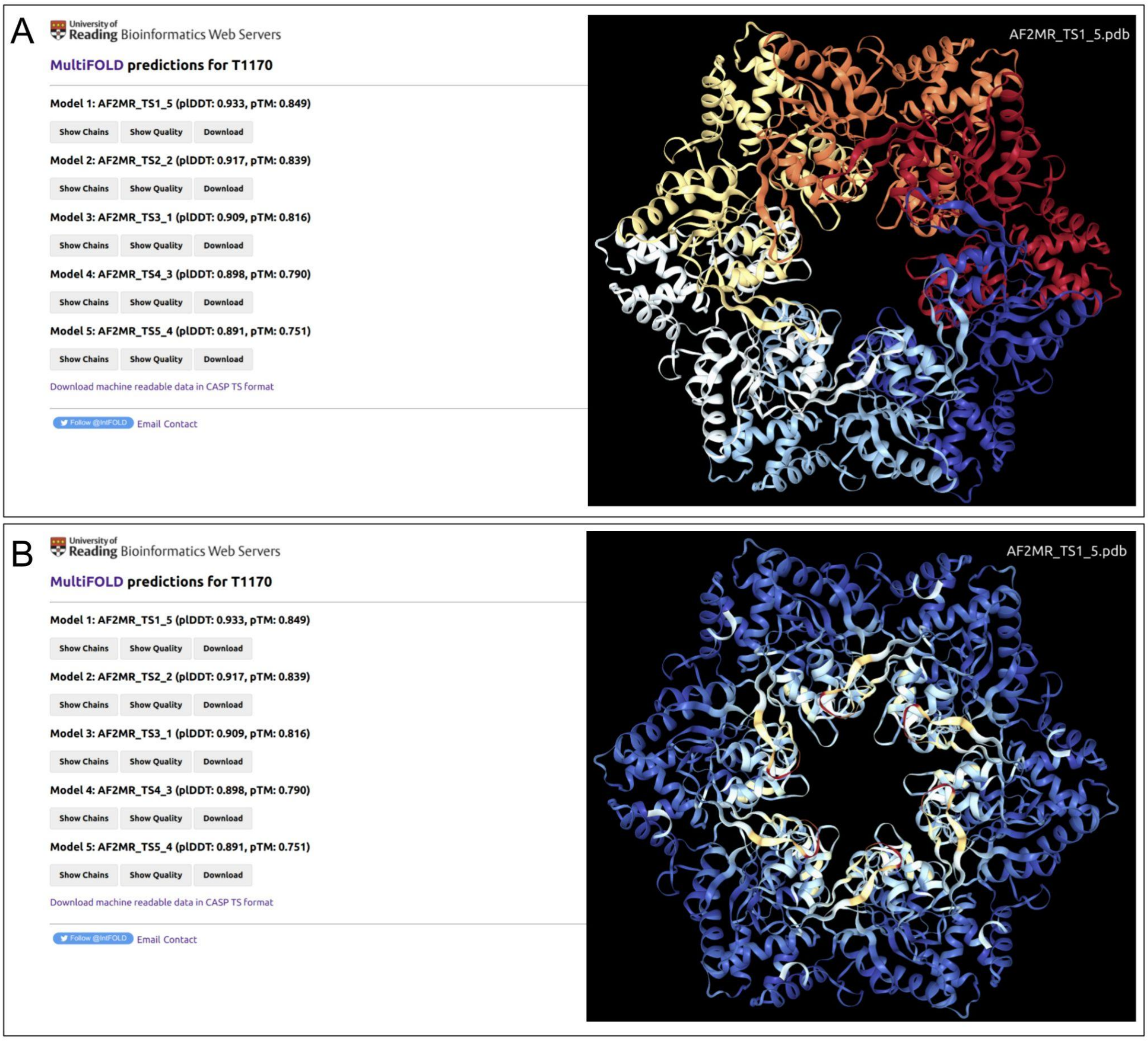

Supplementary Figure 3. ModFOLDdock server results page for CASP15 target H1106 with the stoichiometry A1B1 (only the top 10 models out of 308 are shown in the example screenshots, but users may scroll down to view all of their models). (A) The top-ranked model is coloured by chain identifier which can be selected using the “Show Chains” button. (B) The top model is coloured by predicted local interface model quality (the confidence score relates to the probability of the residue in the model being in the actual interface of the native structure).

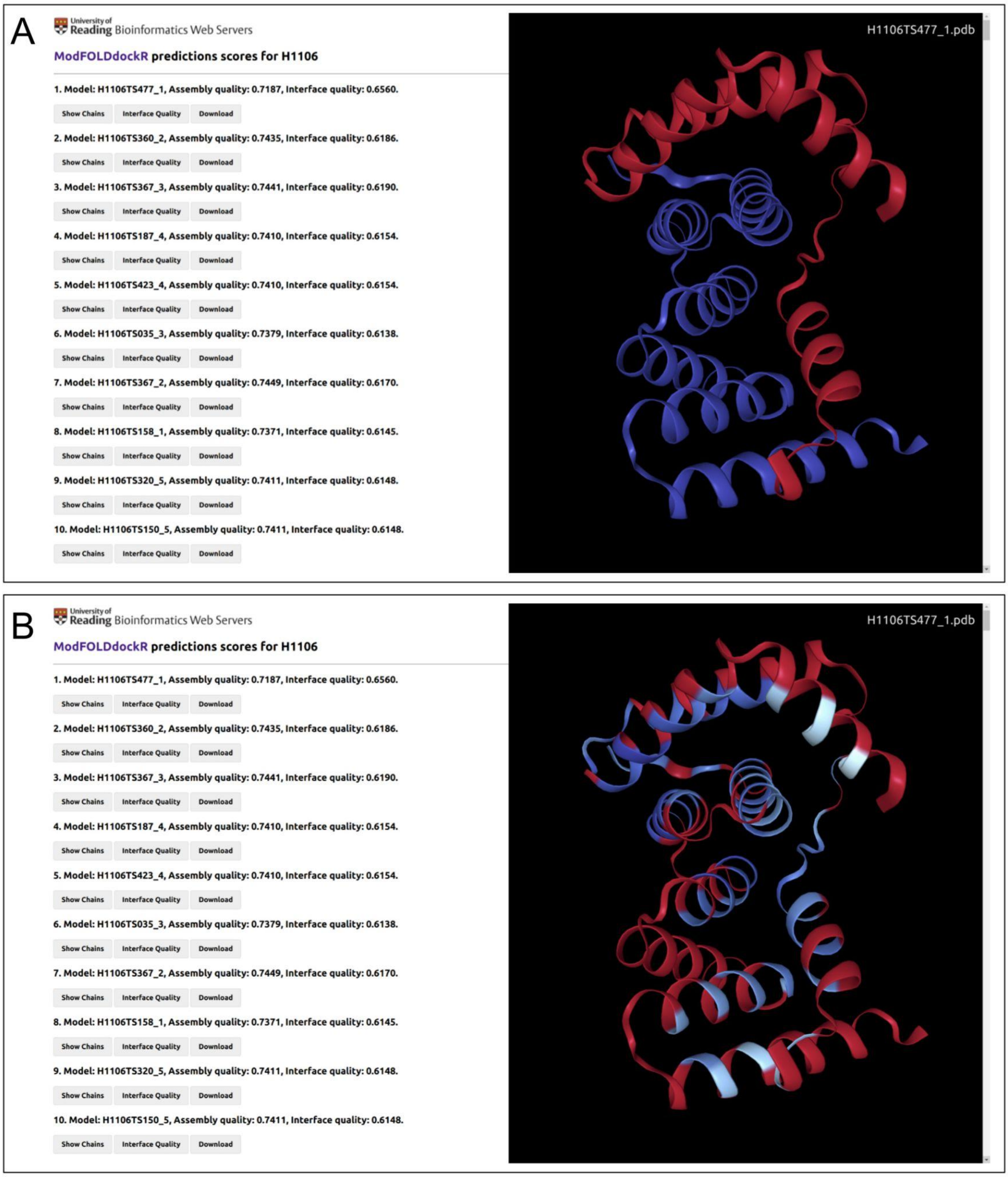

Supplementary Table 1. CAMEO IntFOLD7 - All Targets, Common Subset Comparison (140 targets) - dataset: 1-year [2022-02-04 - 2023-01-28]. Data are from: https://www.cameo3d.org/modeling/.

All models	Avg. IDDT		Avg. CAD-score		Avg. IDDT-BS	
Server Name	Dif.	Best	Dif.	Best	Dif.	Best
AlphaFoldDB 90	-0.39	**82.51**	-0.21	**78.68**	-4.09	**82.6**
AlphaFoldDB 100	-0.35	82.47	-0.26	78.73	-5.61	84.11
**IntFOLD7**	**0**	**82.12**	**0**	**78.47**	**0**	**78.51**
RoseTTAFold	**6.98**	75.14	**5.64**	72.83	**7.86**	70.65
IntFOLD6-TS	**15.93**	66.19	**12.98**	65.49	**8.27**	70.24
IntFOLD5-TS	**17.09**	65.02	**13.48**	64.99	**8.76**	69.75
IntFOLD4-TS	**17.22**	64.89	**14.04**	64.43	**9.74**	68.77
IntFOLD3-TS	**18.99**	63.13	**15.53**	62.94	**10.87**	67.64
SWISS-MODEL	**20.78**	61.34	**17.92**	60.55	**11.9**	66.61
Phyre2	**27.79**	54.33	**23.16**	55.31	**18.05**	60.46

Supplementary Table 2. CASP15 Interdomain Predictions Analysis: Group performance based on combined z-scores. The final ranking was based on the models designated as "1" according to the formula: Z-score(F1) + Z-score(Jaccard score) + Z-score(QS_best). Data are from: https://predictioncenter.org/casp15/zscores_interdomain.cgi.

#	GR name	GR code	Targets Count	SUM Zscore (>0.0)	Rank SUM Zscore (>0.0)	AVG Zscore (>0.0)	Rank AVG Zscore (>0.0)
1	UM-TBM	162	24	37.8507	1	1.5771	1
2	Yang-Server	229	24	27.2108	2	1.1338	3
3	Yang	439	20	19.7115	3	0.9856	4
4	PEZYFoldings	278	15	18.0578	4	1.2039	2
5	server_124	383	24	16.2284	5	0.6762	8
6	Manifold	248	19	14.9308	6	0.7858	5
7	Manifold-E	35	23	14.5759	7	0.6337	10
8	Venclovas	494	19	14.5386	8	0.7652	6
9	DFolding	74	20	13.1098	9	0.6555	9
10	server_126	403	24	12.7975	10	0.5332	16
11	bench	8	20	12.0811	11	0.6041	12
12	BAKER-SERVER	443	24	12.0303	12	0.5013	17
13	DFolding-server	288	19	11.587	13	0.6098	11
**14**	**IntFOLD7**	**151**	**23**	**11.4107**	**14**	**0.4961**	**18**
15	Shennong	466	24	10.7753	15	0.449	23
16	server_123	18	24	10.6511	16	0.4438	24
**17**	**MultiFOLD**	**462**	**22**	**10.6166**	**17**	**0.4826**	**20**

18	RaptorX	166	24	9.9124	18	0.413	27
19	BAKER	185	20	8.862	19	0.4431	25
20	Asclepius	204	19	8.5891	20	0.4521	21
21	Kiharalab_Server	131	24	8.4352	21	0.3515	39
22	UltraFold_Server	125	22	8.2293	22	0.3741	31
23	DFolding-refine	73	19	8.1694	23	0.43	26
24	B11L	208	18	8.0841	24	0.4491	22
25	ManiFold-serv	450	23	7.8702	25	0.3422	42
26	hFold	353	20	7.8019	26	0.3901	28
27	server_122	261	24	7.788	27	0.3245	46
28	DMP	477	14	7.7417	28	0.553	15
29	server_125	264	24	7.6563	29	0.319	47
30	MUFold_H	360	20	7.5603	30	0.378	30
31	OpenFold-SingleSeq	433	19	7.0733	31	0.3723	32
32	OpenFold	441	19	7.0733	31	0.3723	32
33	ShanghaiTech	225	20	7.0584	33	0.3529	36
34	MULTICOM_refine	475	23	6.995	34	0.3041	49
35	Graphen_Medical	97	14	6.8295	35	0.4878	19
36	AP_1	269	20	6.8095	36	0.3405	43
37	Elofsson	320	19	6.6876	37	0.352	37
38	Agemo_mix	92	19	6.6477	38	0.3499	40
39	Panlab	234	20	6.5871	39	0.3294	45
40	McGuffin	180	19	6.5509	40	0.3448	41
41	TRFold	187	17	6.4502	41	0.3794	29
42	BeijingAIProtein	399	17	6.1858	42	0.3639	34
43	UltraFold	54	17	6.1858	42	0.3639	34
44	MULTICOM_deep	158	23	6.1738	44	0.2684	55
45	Agemo	478	17	5.9827	45	0.3519	38
46	FTBiot0119	165	20	5.9501	46	0.2975	51
47	ChaePred	398	20	5.7616	47	0.2881	52
48	WL_team	257	19	5.7417	48	0.3022	50
49	GuijunLab-Assembly	98	23	5.6965	49	0.2477	56
50	trComplex	423	17	5.6135	50	0.3302	44
51	GuijunLab-DeepDA	188	23	5.6052	51	0.2437	57
52	hFold_human	342	18	5.5688	52	0.3094	48
53	Kiharalab	119	20	5.494	53	0.2747	54
54	Seder2022easy	455	19	5.3198	54	0.28	53
55	ColabFold	446	22	5.1993	55	0.2363	60
56	XRC_VU	215	7	4.8243	56	0.6892	7
57	colabfold_human	461	20	4.7985	57	0.2399	58

58	FoldEver	245	23	4.6957	58	0.2042	64
59	GuijunLab-Meta	481	23	4.6656	59	0.2029	65
60	Wallner	37	18	4.1617	60	0.2312	61
61	MULTICOM_qa	86	24	4.111	61	0.1713	68
62	MULTICOM	367	18	3.9011	62	0.2167	62
63	MULTICOM_egnn	120	24	3.8306	63	0.1596	71
64	MULTICOM_human	3	18	3.6862	64	0.2048	63
65	GinobiFold-SER	11	20	3.5591	65	0.178	67
66	GuijunLab-Human	169	19	3.4694	66	0.1826	66
67	NBIS-AF2-standard	270	24	3.3399	67	0.1392	74
68	FoldEver-Hybrid	385	14	3.3126	68	0.2366	59
69	MUFold	298	24	3.3105	69	0.1379	75
70	GuijunLab-Threader	282	22	3.1286	70	0.1422	73
71	GuijunLab-RocketX	89	23	3.06	71	0.133	76
72	Manifold-LC-E	46	5	2.9837	72	0.5967	13
73	GinobiFold	227	17	2.7459	73	0.1615	69
74	Coqualia	434	17	2.7459	73	0.1615	69
75	BhageerathH-Pro	212	18	2.7166	75	0.1509	72
76	Cerebra	315	21	2.5507	76	0.1215	79
77	Bhattacharya	275	19	2.372	77	0.1248	78
78	ShanghaiTech-TS-SER	133	20	2.3314	78	0.1166	80
79	SHT	147	20	2.2983	79	0.1149	81
80	FALCON2	368	24	2.2265	80	0.0928	86
81	FALCON0	333	24	2.2265	80	0.0928	86
82	Pan_Server	219	23	2.161	82	0.094	85
83	hks1988	354	20	2.1535	83	0.1077	82
84	Gonglab-THU	52	17	1.8164	84	0.1068	83
85	DELCLAB	447	18	1.1885	85	0.066	90
86	ESM-single-sequence	67	9	1.1811	86	0.1312	77
87	UNRES	91	14	1.1657	87	0.0833	88
88	QUIC	117	20	1.1187	88	0.0559	93
89	PICNIC	276	20	1.1002	89	0.055	94
90	Seder2022hard	216	10	0.591	90	0.0591	92
91	SHORTLE	64	1	0.59	91	0.59	14
92	wuqi	370	9	0.4176	92	0.0464	95
93	ACOMPMOD	280	5	0.2975	93	0.0595	91
94	MESHI_server	427	2	0.1894	94	0.0947	84
95	EMBER3D	140	10	0.1591	95	0.0159	96
96	Manifold-X	304	1	0.0809	96	0.0809	89
97	RostlabUeFOFold	123	4	0.0346	97	0.0087	97

98	MESHI	362	1	0	98	0	98

Supplementary Table 3. CASP15 Regular Targets Analysis: Z-score based relative group performance. Ranking on the models designated as "1". Server groups on 'all groups' + 'server only' targets. The ranking of groups is based on the sum of Z-scores for the CASP12 formula for TBM domains: GDT_HA + (SphereGrinder + LDDT + CAD_aa)/3 + ASE. Data are from: https://predictioncenter.org/casp15/zscores_final.cgi.

#	GR code	GR name	Domains Count	SUM Zscore (>0.0)	Rank SUM Zscore (>0.0)	AVG Zscore (>0.0)	Rank AVG Zscore (>0.0)
1	162	UM-TBM	62	29.2366	1	0.4716	1
2	298	MUFold	62	25.1375	2	0.4054	3
3	446	ColabFold	62	24.9323	3	0.4021	5
4	475	MULTICOM_refine	62	24.3127	4	0.3921	6
5	120	MULTICOM_egnn	62	24.3073	5	0.3921	7
6	86	MULTICOM_qa	62	23.1819	6	0.3739	8
7	158	MULTICOM_deep	62	23.1201	7	0.3729	9
8	125	UltraFold_Server	62	22.9333	8	0.3699	10
9	35	Manifold-E	62	22.7964	9	0.3677	11
10	188	GuijunLab-DeepDA	62	21.9265	10	0.3537	12
11	288	DFolding-server	62	21.1371	11	0.3409	13
**12**	**151**	**IntFOLD7**	**62**	**21.1239**	**12**	**0.3407**	**15**
13	98	GuijunLab-Assembly	62	21.0867	13	0.3401	16
14	481	GuijunLab-Meta	61	20.7867	14	0.3408	14
15	282	GuijunLab-Threader	62	20.7119	15	0.3341	17
16	166	RaptorX	62	19.9717	16	0.3221	18
17	383	server_124	62	19.6794	17	0.3174	20
18	353	hFold	61	19.5899	18	0.3211	19
19	245	FoldEver	62	19.1977	19	0.3096	22
**20**	**462**	**MultiFOLD**	**62**	**19.0098**	**20**	**0.3066**	**23**
21	131	Kiharalab_Server	62	18.985	21	0.3062	24
22	18	server_123	62	18.984	22	0.3062	25
23	229	Yang-Server	62	18.8055	23	0.3033	26
24	270	NBIS-AF2-standard	62	18.6993	24	0.3016	27
25	450	ManiFold-serv	62	18.1488	25	0.2927	30
26	466	Shennong	58	18.104	26	0.3121	21
27	261	server_122	62	18.015	27	0.2906	31
28	264	server_125	62	18.0085	28	0.2905	32
29	89	GuijunLab-RocketX	62	17.7738	29	0.2867	33
30	403	server_126	62	16.3719	30	0.2641	34
31	390	NBIS-AF2-multimer	32	12.8713	31	0.4022	4

32	215	XRC_VU	43	12.7099	32	0.2956	29
33	71	RaptorX-Multimer	29	11.8746	33	0.4095	2
34	133	ShanghaiTech-TS-SER	59	11.2284	34	0.1903	36
35	443	BAKER-SERVER	62	11.0783	35	0.1787	38
36	11	GinobiFold-SER	59	10.6223	36	0.18	37
37	239	Yang-Multimer	29	8.7329	37	0.3011	28
38	73	DFolding-refine	61	7.5166	38	0.1232	39
39	219	Pan_Server	59	3.7489	39	0.0635	40
40	46	Manifold-LC-E	11	2.2158	40	0.2014	35
41	370	wuqi	49	1.8982	41	0.0387	41
42	427	MESHI_server	46	1.2265	42	0.0267	42
43	280	ACOMPMOD	46	0.8157	43	0.0177	43
44	368	FALCON2	61	0.5078	44	0.0083	44
45	333	FALCON0	61	0.5078	44	0.0083	44
46	212	BhageerathH-Pro	56	0.1234	46	0.0022	46
47	315	Cerebra	62	0.0171	47	0.0003	47

Supplementary Table 4. CASP15 Regular Targets Analysis: Z-score based relative group performance. Ranking on the models designated as "1". Server groups on 'all groups' + 'server only' targets. The ranking of groups is based on the sum of Z-scores for the CASP15 formula for ALL domains: 1/6*(GDT_HA + reLLG_lddt + ASE) + 1/16*(LDDT + CAD_aa + SG + SC_error) + 1/12*(MolProbity + BB_error + DipDiff). Data are from: https://predictioncenter.org/casp15/zscores_final.cgi.

#	GR code	GR name	Domains Count	SUM Zscore (>0.0)	Rank SUM Zscore (>0.0)	AVG Zscore (>0.0)	Rank AVG Zscore (>0.0)
1	162	UM-TBM	109	67.2002	1	0.6165	1
2	229	Yang-Server	108	61.9838	2	0.5739	2
3	475	MULTICOM_refine	109	46.7553	3	0.4289	5
4	35	Manifold-E	109	46.5604	4	0.4272	6
5	158	MULTICOM_deep	109	44.5019	5	0.4083	8
6	86	MULTICOM_qa	109	44.1312	6	0.4049	9
7	120	MULTICOM_egnn	109	44.1113	7	0.4047	10
8	446	ColabFold	109	42.4951	8	0.3899	11
9	288	DFolding-server	109	40.3075	9	0.3698	12
10	298	MUFold	109	39.7763	10	0.3649	13
11	125	UltraFold_Server	109	38.6993	11	0.355	14
12	166	RaptorX	109	38.1597	12	0.3501	15
13	443	BAKER-SERVER	109	36.4303	13	0.3342	17
14	188	GuijunLab-DeepDA	109	36.362	14	0.3336	18
15	466	Shennong	105	35.6774	15	0.3398	16
16	131	Kiharalab_Server	109	35.1998	16	0.3229	19
**17**	**462**	**MultiFOLD**	**109**	**35.081**	**17**	**0.3218**	**20**

18	98	GuijunLab-Assembly	109	34.4453	18	0.316	21
**19**	**151**	**IntFOLD7**	**109**	**34.2882**	**19**	**0.3146**	**22**
20	282	GuijunLab-Threader	109	33.7074	20	0.3092	24
21	383	server_124	109	33.3507	21	0.306	25
22	353	hFold	106	32.986	22	0.3112	23
23	245	FoldEver	109	32.2247	23	0.2956	28
24	270	NBIS-AF2-standard	109	32.0511	24	0.294	29
25	481	GuijunLab-Meta	107	31.7189	25	0.2964	27
26	261	server_122	109	30.5781	26	0.2805	30
27	18	server_123	109	30.3196	27	0.2782	31
28	403	server_126	109	30.1623	28	0.2767	32
29	264	server_125	109	30.1061	29	0.2762	33
30	450	ManiFold-serv	109	28.1847	30	0.2586	35
31	89	GuijunLab-RocketX	108	28.0519	31	0.2597	34
32	133	ShanghaiTech-TS-SER	105	24.1274	32	0.2298	36
33	215	XRC_VU	80	23.7588	33	0.297	26
34	239	Yang-Multimer	45	21.2334	34	0.4719	3
35	390	NBIS-AF2-multimer	50	20.7387	35	0.4148	7
36	11	GinobiFold-SER	105	20.0288	36	0.1908	38
37	71	RaptorX-Multimer	45	19.7221	37	0.4383	4
38	73	DFolding-refine	106	13.644	38	0.1287	39
39	370	wuqi	87	8.1179	39	0.0933	40
40	280	ACOMPMOD	78	3.6999	40	0.0474	41
41	219	Pan_Server	104	3.0839	41	0.0297	42
42	46	Manifold-LC-E	15	2.9338	42	0.1956	37
43	368	FALCON2	107	2.2185	43	0.0207	44
44	333	FALCON0	107	2.2086	44	0.0206	45
45	427	MESHI_server	76	2.1152	45	0.0278	43
46	315	Cerebra	109	1.0481	46	0.0096	46
47	212	BhageerathH-Pro	103	0.6155	47	0.006	47

Supplementary Table 5. CASP15 Regular Targets Analysis: Z-score based relative group performance. Ranking on the models designated as "1". Server groups on 'all groups' + 'server only' targets. The ranking of groups is based on the sum of Z-scores for GDT_TS. Data are from: https://predictioncenter.org/casp15/zscores_final.cgi.

#	GR code	GR name	Domains Count	SUM Zscore (>0.0)	Rank SUM Zscore (>0.0)	AVG Zscore (>0.0)	Rank AVG Zscore (>0.0)
1	229	Yang-Server	108	92.5982	1	0.8574	1
2	162	UM-TBM	109	91.841	2	0.8426	2
3	35	Manifold-E	109	63.2197	3	0.58	4
4	475	MULTICOM_refine	109	54.8979	4	0.5037	6
5	120	MULTICOM_egnn	109	52.848	5	0.4848	7
6	158	MULTICOM_deep	109	51.0686	6	0.4685	8

7	288	DFolding-server	109	49.9465	7	0.4582	10
8	86	MULTICOM_qa	109	49.494	8	0.4541	11
**9**	**462**	**MultiFOLD**	**109**	**47.2831**	**9**	**0.4338**	**12**
10	446	ColabFold	109	46.8624	10	0.4299	13
11	166	RaptorX	109	45.809	11	0.4203	14
12	125	UltraFold_Server	109	40.3241	12	0.3699	15
13	298	MUFold	109	39.7967	13	0.3651	16
14	131	Kiharalab_Server	109	39.5082	14	0.3625	17
15	98	GuijunLab-Assembly	109	36.6711	15	0.3364	19
16	188	GuijunLab-DeepDA	109	36.5916	16	0.3357	20
17	466	Shennong	105	35.9139	17	0.342	18
18	383	server_124	109	35.5967	18	0.3266	21
19	403	server_126	109	35.532	19	0.326	22
20	270	NBIS-AF2-standard	109	34.1607	20	0.3134	24
21	245	FoldEver	109	33.8134	21	0.3102	25
22	353	hFold	106	33.3984	22	0.3151	23
**23**	**151**	**IntFOLD7**	**109**	**32.9872**	**23**	**0.3026**	**26**
24	18	server_123	109	31.1801	24	0.2861	28
25	261	server_122	109	30.8276	25	0.2828	29
26	264	server_125	109	30.2311	26	0.2773	30
27	481	GuijunLab-Meta	107	29.1492	27	0.2724	31
28	282	GuijunLab-Threader	109	28.1772	28	0.2585	32
29	239	Yang-Multimer	45	27.4611	29	0.6102	3
30	89	GuijunLab-RocketX	108	27.3532	30	0.2533	33
31	73	DFolding-refine	106	25.3667	31	0.2393	35
32	133	ShanghaiTech-TS-SER	105	25.2661	32	0.2406	34
33	11	GinobiFold-SER	105	24.608	33	0.2344	36
34	215	XRC_VU	80	23.5756	34	0.2947	27
35	71	RaptorX-Multimer	45	23.483	35	0.5218	5
36	450	ManiFold-serv	109	23.0836	36	0.2118	37
37	390	NBIS-AF2-multimer	50	23.0656	37	0.4613	9
38	443	BAKER-SERVER	109	20.948	38	0.1922	38
39	427	MESHI_server	76	8.612	39	0.1133	40
40	219	Pan_Server	104	4.7695	40	0.0459	41
41	315	Cerebra	109	2.927	41	0.0269	43
42	370	wuqi	87	2.7157	42	0.0312	42
43	46	Manifold-LC-E	15	2.4712	43	0.1647	39
44	368	FALCON2	107	2.3896	44	0.0223	44
45	333	FALCON0	107	2.3896	44	0.0223	44
46	212	BhageerathH-Pro	103	1.2395	46	0.012	46
47	280	ACOMPMOD	78	0.1265	47	0.0016	47

Supplementary Table 6. CASP15 Multimer Targets Analysis: Server group performance based on combined z-scores (human groups have been removed). The ranking of groups is based on the sum of Z-scores for the models designated as "1". Assessors' formula: Z-score(ICS) + Z-score(IPS) + Z-score(LDDTo) + Z-score(TM). ICS: Interface Contact Score (a.k.a. F1 score). IPS: Interface Patch Score (a.k.a. Jaccard coefficient)". Data are from: https://predictioncenter.org/casp15/zscores_multimer.cgi.

#	GR name	GR code	Targets Count	SUM Zscore (>0.0)	Rank SUM Zscore (>0.0)	AVG Zscore (>0.0)	Rank AVG Zscore (>0.0)
1	Yang-Multimer	239	41	25.0147	1	0.6101	1
2	Manifold-E	35	43	19.864	2	0.462	4
3	MULTICOM_qa	86	43	19.6209	3	0.4563	5
4	MULTICOM_deep	158	43	17.5549	4	0.4083	7
5	DFolding-server	288	35	17.4518	5	0.4986	3
6	Kiharalab_Server	131	42	16.5675	6	0.3945	9
7	UltraFold_Server	125	43	15.7925	7	0.3673	10
**8**	**MultiFOLD**	**462**	**42**	**15.353**	**8**	**0.3655**	**11**
9	MUFold	298	43	14.0905	9	0.3277	13
10	ColabFold	446	41	13.1542	10	0.3208	14
11	NBIS-AF2-multimer	390	43	12.2984	11	0.286	16
12	RaptorX-Multimer	71	42	12.076	12	0.2875	15
13	GuijunLab-Assembly	98	43	10.4977	13	0.2441	19
14	Yang-Server	229	21	10.495	14	0.4998	2
15	DFolding-refine	73	37	9.7678	15	0.264	18
16	GinobiFold-SER	11	17	6.7751	16	0.3985	8
17	GuijunLab-DeepDA	188	42	6.5885	17	0.1569	20
18	ShanghaiTech-TS-SER	133	17	4.721	18	0.2777	17
19	FoldEver	245	31	4.5593	19	0.1471	21
20	Manifold-LC-E	46	9	4.0502	20	0.45	6
21	XRC_VU	215	14	1.1453	21	0.0818	22
22	Cerebra	315	8	0.5344	22	0.0668	23
23	GuijunLab-Meta	481	1	0.3431	23	0.3431	12
24	FALCON0	333	20	0	24	0	24
25	FALCON2	368	20	0	25	0	25
26	wuqi	370	1	0	26	0	26

Supplementary Figure 4. Examples of some of the top MultiFOLD quaternary structure predictions from CASP15 compared with the native structures. The MultiFOLD models are shown in the left columns and coloured by chain ID. The native quaternary structures are in the middle columns, also coloured by chain ID. The right columns show the superpositions of the MultiFOLD models (green) and native quaternary structures (cyan). (A) T1124 homo-2-mer, QS=0.918, Oligo-lDDT=0.874. (B) T1132 homo-6-mer, QS=0.953, Oligo-lDDT=0.932. (C) H1151 hetero-2-mer, QS=0.910, Oligo-lDDT=0.852. (D) H1157 hetero-2-mer, QS=0.791, Oligo-lDDT=0.708. (E) H1167 hetero-3-mer, QS=0.688, Oligo-lDDT=0.843. (F) T1187 homo-2-mer, QS=0.888, Oligo-lDDT=0.850.

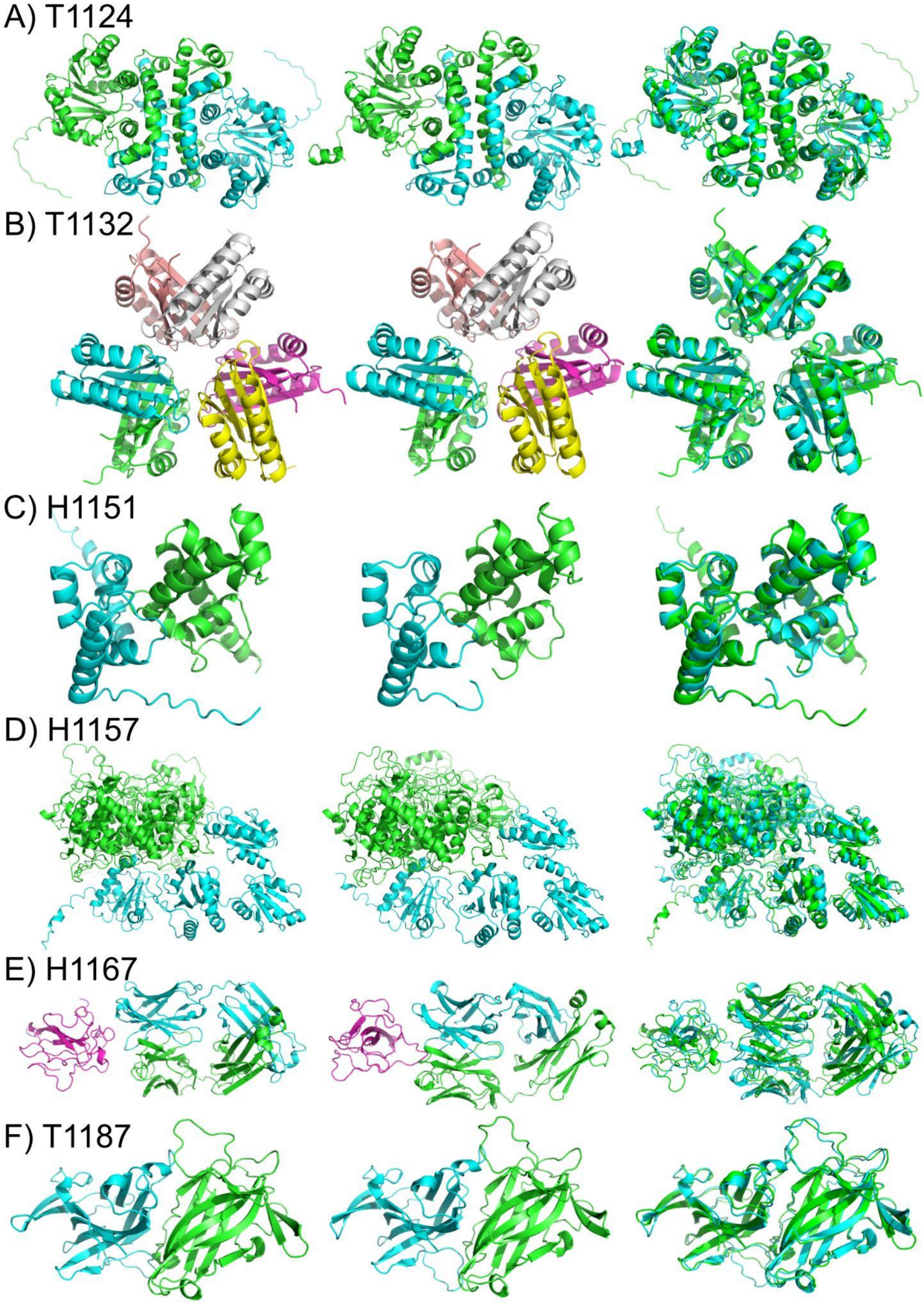
A) T1124
B) T1132
C) H1151
D) H1157
E) H1167
F) T1187

Supplementary Table 7. Official CASP15 EMA Analysis - Global (fold). The table is ranked by the group performance based on combined assessor-based formula (0.5*Pearson(GDT_TS) + 0.5*Spearman(GDT_TS) + AUC(GDT_TS) - Loss(GDT_TS) + 0.5*Pearson(TM) + 0.5*Spearman(TM) + AUC(TM) - Loss(TM)). GR# is the group number. AUC is the area under the curve of the Receiver Operating Characteristic (ROC). Data are from: https://predictioncenter.org/casp15/qa_local.cgi

		GDT_TS-like				TM				
**Group Name**	**GR#**	**Pearson**	**Spearman**	**AUC**	**Loss**	**Pearson**	**Spearman**	**AUC**	**Loss**	**Assessor based formula**
MULTICOM_qa	86	0.629	0.559	0.689	0.291	0.712	0.58	0.703	0.152	2.189
AssemblyConsensus	0	0.643	0.577	0.688	0.306	0.635	0.585	0.706	0.177	2.131
ModFOLDdock	41	0.613	0.487	0.677	0.258	0.636	0.517	0.684	0.127	2.1025
ModFOLDdockR	266	0.565	0.51	0.679	0.286	0.635	0.504	0.679	0.142	2.037
Venclovas	494	0.53	0.435	0.676	0.216	0.49	0.437	0.68	0.123	1.963
Manifold	248	0.517	0.497	0.663	0.295	0.541	0.51	0.664	0.146	1.9185
GuijunLab-Threader	282	0.573	0.468	0.654	0.376	0.633	0.493	0.668	0.207	1.8225
GuijunLab-Human	169	0.54	0.463	0.652	0.35	0.601	0.459	0.64	0.189	1.7845
VoroIF	121	0.492	0.345	0.64	0.271	0.483	0.351	0.641	0.16	1.6855
Bhattacharya	275	0.47	0.419	0.647	0.344	0.474	0.423	0.655	0.195	1.656
VoroMQA-select-2020	2	0.427	0.384	0.652	0.315	0.414	0.394	0.663	0.179	1.6305
ModFOLDdockS	83	0.513	0.403	0.632	0.372	0.551	0.434	0.645	0.227	1.6285
GuijunLab-RocketX	89	0.402	0.335	0.652	0.308	0.464	0.361	0.655	0.165	1.615
MUFold	298	0.517	0.364	0.611	0.389	0.625	0.409	0.617	0.24	1.5565
GuijunLab-Assembly	98	0.39	0.368	0.634	0.348	0.437	0.373	0.63	0.193	1.507
MUFold2	283	0.473	0.339	0.605	0.408	0.564	0.351	0.604	0.239	1.4255
ChaePred	398	0.414	0.329	0.639	0.412	0.453	0.327	0.651	0.233	1.4065
MULTICOM_egnn	120	0.26	0.275	0.611	0.365	0.257	0.257	0.607	0.201	1.1765
FoldEver	245	0.326	0.254	0.596	0.453	0.381	0.247	0.594	0.267	1.074
LAW	426	0.267	0.219	0.589	0.458	0.332	0.215	0.595	0.305	0.9375

MASS	468	0.214	0.17	0.565	0.416	0.269	0.165	0.566	0.259	0.865
MULTICOM_deep	158	0.193	0.191	0.559	0.443	0.183	0.176	0.561	0.299	0.7495
APOLLO	168	0.055	0.049	0.507	0.401	0.059	0.056	0.51	0.247	0.4785

Supplementary Table 8. Official CASP15 EMA Analysis - Global (Interface). The table is ranked by the group performance based on combined assessor-based formula (0.5*Pearson(DockQ-wave) + 0.5*Spearman(DockQ-wave) + AUC(DockQ-wave) - Loss(DockQ-wave) + 0.5*Pearson(QS) + 0.5*Spearman(QS) + AUC(QS) - Loss(QS)). GR# is the group number. AUC is the area under the curve of the Receiver Operating Characteristic (ROC). Data are from: https://predictioncenter.org/casp15/qa_local.cgi

		DockQ-wave				QS				Assessor based formula
**Group Name**	**GR#**	**Pearson**	**Spearman**	**AUC**	**Loss**	**Pearson**	**Spearman**	**AUC**	**Loss**	
AssemblyConsensus	0	0.685	0.591	0.703	0.25	0.765	0.658	0.734	0.224	2.3125
ModFOLDdockR	266	0.624	0.548	0.694	0.223	0.673	0.577	0.695	0.213	2.164
ModFOLDdock	41	0.628	0.55	0.691	0.236	0.673	0.58	0.692	0.22	2.1425
ModFOLDdockS	83	0.549	0.492	0.668	0.296	0.603	0.535	0.688	0.298	1.8515
VoroIF	121	0.535	0.379	0.66	0.234	0.563	0.405	0.668	0.237	1.798
Venclovas	494	0.54	0.392	0.658	0.22	0.552	0.383	0.654	0.24	1.7855
VoroMQA-select-2020	2	0.456	0.381	0.675	0.209	0.503	0.372	0.668	0.208	1.782
GuijunLab-RocketX	89	0.481	0.377	0.665	0.235	0.532	0.427	0.668	0.261	1.7455
ChaePred	398	0.488	0.397	0.676	0.34	0.515	0.366	0.663	0.374	1.508
Manifold	248	0.463	0.378	0.615	0.342	0.534	0.46	0.649	0.355	1.4845
FoldEver	245	0.397	0.309	0.647	0.426	0.393	0.298	0.634	0.502	1.0515
MULTICOM_qa	86	0.356	0.302	0.572	0.462	0.416	0.328	0.578	0.523	0.866
MASS	468	0.27	0.213	0.581	0.416	0.301	0.202	0.572	0.433	0.797
DLA-Ranker	101	0.342	0.263	0.61	0.483	0.355	0.281	0.588	0.592	0.7435
Bhattacharya	275	0.265	0.232	0.541	0.542	0.307	0.259	0.555	0.611	0.4745
MULTICOM_deep	158	0.218	0.206	0.54	0.492	0.241	0.215	0.548	0.574	0.462
APOLLO	168	0.206	0.195	0.547	0.491	0.198	0.176	0.548	0.571	0.4205
LAW	426	0.201	0.195	0.559	0.608	0.242	0.198	0.549	0.752	0.166

Supplementary Table 9. Official CASP15 EMA Analysis - Local mode. The table is ranked by the group performance based on combined assessor-based formula (0.5*Pearson(PatchDockQ) + 0.5*Spearman(PatchDockQ) + AUC(PatchDockQ) + 0.5*Pearson(PatchQS) + 0.5*Spearman(PatchQS) + AUC(PatchQS) +0.5*Pearson(CAD) + 0.5*Spearman(CAD) + AUC(CAD) + 0.5*Pearson(LDDT) + 0.5*Spearman(LDDT) + AUC(LDDT)). GR# is the group number. AUC is the area under the curve of the Receiver Operating Characteristic (ROC). Data are from: https://predictioncenter.org/casp15/qa_local.cgi

Group Name	GR#	PatchDockQ			PatchQS			CAD			LDDT			Assessor based formula
		Pearson	Spearman	AUC	Pearson	Spearman	AUC	Pearson	Spearman	AUC	Pearson	Spearman	AUC	
GuijunLab-RocketX	89	0.432	0.406	0.698	0.41	0.401	0.701	0.505	0.456	0.714	0.564	0.535	0.755	4.7225
ModFOLDdockR	266	0.45	0.393	0.652	0.46	0.372	0.651	0.411	0.369	0.651	0.476	0.433	0.681	4.317
ModFOLDdockS	83	0.388	0.339	0.635	0.382	0.323	0.631	0.42	0.379	0.66	0.455	0.416	0.674	4.151
VoroIF	121	0.415	0.393	0.675	0.39	0.399	0.683	0.272	0.271	0.619	0.333	0.339	0.664	4.047
Venclovas	494	0.415	0.392	0.675	0.389	0.399	0.683	0.271	0.271	0.619	0.332	0.338	0.664	4.0445
ModFOLDdock	41	0.36	0.306	0.604	0.393	0.3	0.608	0.209	0.2	0.572	0.243	0.227	0.584	3.487
FoldEver	245	0.209	0.207	0.591	0.195	0.212	0.604	0.217	0.194	0.583	0.277	0.279	0.625	3.298
Manifold	248	0.302	0.271	0.567	0.315	0.266	0.567	0.153	0.149	0.529	0.18	0.176	0.542	3.111
APOLLO	168	0.2	0.217	0.559	0.192	0.215	0.567	0.156	0.159	0.549	0.192	0.213	0.565	3.012
MASS	468	0.144	0.176	0.533	0.123	0.168	0.529	0.141	0.152	0.521	0.151	0.172	0.527	2.7235
LAW	426	0.096	0.101	0.53	0.093	0.101	0.526	0.143	0.133	0.513	0.169	0.168	0.525	2.596
MULTICOM_deep	158	0.104	0.116	0.546	0.115	0.134	0.55	0.082	0.091	0.534	0.091	0.094	0.538	2.5815
DLA-Ranker	101	0.097	0.123	0.531	0.092	0.109	0.524	0.093	0.095	0.526	0.1	0.112	0.529	2.5205
